# Improving patient satisfaction based on service quality in clinical trials: A cross-sectional study

**DOI:** 10.1371/journal.pone.0313340

**Published:** 2024-12-27

**Authors:** Go-Eun Lee, Sue Kim, Sang Hui Chu, Jeong-Ho Seok, So Yoon Kim, Sanghee Kim

**Affiliations:** 1 College of Nursing, Yonsei University, Seoul, Republic of Korea; 2 Mo-Im Kim Nursing Research Institute, Yonsei University, Seoul, Republic of Korea; 3 Department of Psychiatry, Gangnam Severance Hospital, Seoul, Republic of Korea; 4 Institute of Behavioral Sciences in Medicine, College of Medicine, Yonsei University, Seoul, Republic of Korea; 5 Department of Medical Humanities and Social Sciences College of Medicine, College of Medicine, Yonsei University, Seoul, Republic of Korea; 6 Asian Institute for Bioethics and Health Law, Yonsei University, Seoul, Republic of Korea; Universiti Malaya, MALAYSIA

## Abstract

**Background:**

Participants’ satisfaction is an important factor in securing competitiveness in clinical trials. In many industries, such as healthcare, customer service quality has been analyzed to increase customer satisfaction. However, no study so far has attempted to measure participants’ perceptions of service quality in the clinical trial area and identify its effect on participant satisfaction.

**Objective:**

This study examined the experiences and perceptions of clinical trial participants in terms of service quality and identified the factors that impact participant satisfaction in clinical trials.

**Methods:**

This study used a cross-sectional descriptive and explanatory research design. Data were collected from March 29 to May 26, 2023, via a survey. The survey was conducted with 206 adults participating in clinical trials at two hospitals in Korea. The collected data were analyzed using descriptive statistics, independent t-tests, one-way ANOVA, Pearson’s correlation, and multiple linear regression analysis.

**Results:**

Participants’ perceptions of the service quality and their satisfaction in clinical trials were generally positive. The variables that significantly predicted participant satisfaction in clinical trials included quality of interaction with researchers, physical environment, performance results in clinical trials, changes in health status after participating in the trial, and consideration of discontinuing the trial.

**Conclusions:**

Participants’ perception of the service quality significantly affected their satisfaction in clinical trials. Thus, all components of service quality should be considered in the overall clinical trial process to increase participants’ satisfaction.

## Introduction

Clinical trials play a vital role in advancing medical technology by providing essential evidence regarding the efficacy and safety of new treatments [1]. As of February 2024, there have been a total of 482,906 clinical trials registered on Clinicaltrials.gov, a clinical trial registry operated by the National Institutes of Health (NIH) in the United States, since the year 2000 [2]. This indicates that a significant number of individuals, including healthy volunteers and patients, are participating in clinical trials globally, as human participants are essential for conducting clinical trials. As the clinical trial market grows quantitatively, there has been an increasing focus on participant satisfaction beyond ensuring the basic rights of clinical trial participants as a way to enhance competitiveness in the global clinical trial market [3].

Participant satisfaction plays a vital role in increasing compliance with the clinical trial protocol and in promoting the continuation of participation in trials. Ultimately, it is a significant variable that affects both the progress and outcomes of trials [4, 5]. Furthermore, high participant satisfaction is instrumental in fostering competitiveness, promoting not only a positive perception of clinical trials but also encouraging participation in other trials [6].

In this regard, previous studies have assessed participant satisfaction in clinical trials, both domestically and internationally. Most clinical trial participants have reported satisfaction with their trial participation [5–9], with factors such as the participant-researcher relationship, trust, and interaction having a greater impact than research-related factors such as the purpose and methodology of the study [3, 5, 10, 11]. Various other factors, including the informed consent process, participants’ level of understanding of the trial [3, 7, 10, 12], and trial procedures [5, 10, 12] have been found to be associated with participant satisfaction in clinical trials.

However, most studies have predominantly focused on certain fragmented elements considered important in clinical trials, such as the informed consent process, rather than on systematically examining the overall experience of clinical trial participants and evaluating their satisfaction with the entire process [10, 13]. Furthermore, there are limited systematic analyses of factors influencing participant satisfaction in clinical trials and attempts to improve participant satisfaction through such analysis [4]. This contrasts with decades of standardized and validated surveys measuring patient experiences with clinical treatment in clinical settings, leading to various successful programs aimed at improving healthcare environments [13].

Meanwhile, as positive customer perception of received services directly translates into customer satisfaction, service quality is considered a prerequisite for customer satisfaction. Accordingly, various service industries, including healthcare, have devoted considerable effort to the multidimensional measurement of service quality provided to customers from the consumer’s perspective rather than the service provider, aiming to improve customer satisfaction [14, 15]. Patients’ perceived medical service quality has been shown to increase patient satisfaction [14, 16, 17] and loyalty [18] to medical institutions, ultimately leading to increased willingness to revisit [14].

While the emphasis on the quality of clinical trials has increased, the focus has predominantly been on provider-centered features, such as trial design, completeness of outcomes, and procedural rigor [19]. To the best of our knowledge, no prior attempts have been made to analyze the quality of clinical trials in terms of services provided to participants, thereby increasing participant satisfaction. Therefore, this study comprehensively investigated the experiences and perceptions of the service quality of clinical trials among Korean clinical trial participants, and identified the factors that influence satisfaction with clinical trial participation in terms of clinical trial service quality. By doing so, we seek to contribute to optimizing the experience of clinical trial participants. A survey was conducted with 206 adults participating in clinical trials at two hospitals in Korea. The results suggested that participants’ perception of the service quality significantly affects their satisfaction in clinical trials. Variables, such as quality of interaction with researchers, physical environment, and performance results in clinical trials, significantly predicted participant satisfaction in clinical trials.

## Materials and methods

### Study design

This study used a cross-sectional descriptive and explanatory research design to identify perceptions of the service quality and satisfaction among participants in clinical trials.

### Conceptual framework of the study

The conceptual framework of this study is based on Brady and Cronin’s [20] hierarchical model of service quality, which comprises three primary dimensions: interaction, physical environment, and outcome quality. Interaction quality is the functional service quality representing the interaction between service providers and customers in the service delivery process. Physical environment quality refers to tangible elements of service, such as physical facilities and equipment for service provision. Outcome quality, defined as what customers obtain after receiving the service from the provider, comprises resultant factors such as waiting time, tangible evidence, and valence (favorability). We adapted this model to the context of clinical trials:

- Interaction quality: quality of interaction with researchers in clinical trials
- Physical environment quality: quality of physical environment in clinical trials
- Outcome quality: quality of performance in clinical trials

**Fig 1:**
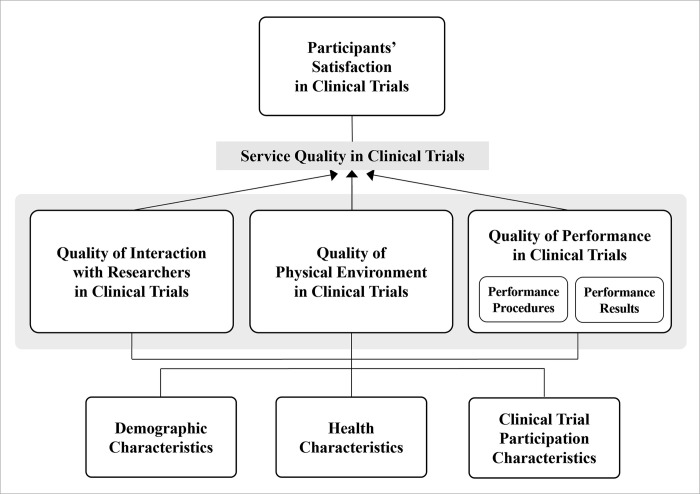
Conceptual framework of the study.

Furthermore, previous studies using Brady and Cronin’s model have provided evidence of the significant impact of healthcare service quality on patient satisfaction [21, 22]. Although no research has yet been conducted to examine the relationship between service quality and participant satisfaction in clinical trials, components we have proposed as elements of clinical trial service quality—such as interaction with the researchers and trial procedures—were associated with participant satisfaction in clinical trials [3, 5, 7, 10–12]. Based on these findings, we aimed to construct dimensions of clinical trial service quality using Brady and Cronin’s model and examine their impact on participant satisfaction (Fig 1).

### Participants

The target population of this study was adults (≥ 19 years) who were either participating or had participated in drug or medical device clinical trials in Korea. Eligible participants for this study were recruited from two hospitals under Y Medical Center in Seoul using convenience sampling. The sample size was calculated using G*Power software (version 3.1.9.7). When applying a significance level (α) of .05, a power (1-β) of .80, a medium effect size of .15, and 29 independent variables, the minimum sample size required for multiple regression analysis is 184 [23]. Accordingly, the target sample size was planned to be 221, considering a dropout rate of 20%. A total of 219 trial participants agreed to participate in this study, of whom 13 did not complete the survey. Excluding these responses, 206 responses (94.1% of the total participants) were found to be suitable. Hence, the recruitment of participants was discontinued, and the results were analyzed.

### Instruments

The research instrument consisted of a structured questionnaire comprising 95 items, including 8 items on participant satisfaction in clinical trials, 58 on service quality in clinical trials, 18 on clinical trial participant characteristics, 7 on demographic characteristics, and 4 on health-related characteristics (Table 1). To measure the quality of interaction with research staff and the quality of clinical trial execution, we adapted and translated existing tools in accordance with the WHO’s Process of Translation and Adaptation of Instruments [24]. However, due to the absence of a pre-existing instrument for assessing the quality of the clinical trial environment, we developed new items based on a literature review and individual interviews. The initial draft of the questionnaire, comprising both translated items and newly developed ones, were finalized through content validity testing by an expert panel and item review by clinical trial participants. Prior to analyzing the survey results, we conducted reliability and validity tests on the finalized items to ensure that the psychometric properties of the survey were acceptable. The specific process for the development of items for this study has been reported previously [25].

**Table 1:** Instruments.

Concepts		Instruments (Author)	Sub-domains/Variables	Items	Cronbach’s α	
					Original	This study
Service quality in clinical trials	Quality of interaction with researchers	RPPS [13]	Information, education, and communication (7)	17	.66 –.83	.896
			Trust (6)			
			Respect for participant preferences (4)			
	Quality of physical environment	The developed by researchers	Securing facilities and space (4)	9	-	.884
			Convenience (5)			
	Quality of performance procedures	RPPS [13]	Informed consent (12)	18	.72 –.84	.845
			Coordination of care (6)			
	Quality of performance results	QUIC [26]	Subjective understanding of clinical trials	14	.77 ^1)^	.900
Participant satisfaction in clinical trials		RSS [8]		8	-	.900
Demographic characteristics			Gender, age, marital and employment status, education level, monthly family income level, and financial burden about meeting medical expenses	7		
Health characteristics			Clinical trial-related diseases, duration of illness, number of comorbidities, and participants’ subjective health status.	4		
Clinical trial participation characteristics			The number of instances of clinical trial participation, type of clinical trial, participation duration, primary motivation, reasons for participation, procedure for obtaining informed consent, experience of adverse events and treatment efficacy, considerations for discontinuing participation, reasons for discontinuation or continuation of participation, and factors considered important for future clinical trials	18		

RPPS, Research Participant Perception Survey; QUIC, Quality of Informed Consent; RSS, Research Satisfaction Survey

^1)^ Intraclass Correlations Coefficient (ICC)

#### Participant satisfaction in clinical trials.

Participant satisfaction in clinical trials was assessed using the modified Korean version of the Research Satisfaction Survey (RSS). The original RSS was developed by Sano et al. [8] to assess participant satisfaction in dementia prevention research. We modified the original version to assess participants’ satisfaction with clinical trials. The modified Korean RSS comprised 8 items that assessed overall satisfaction with clinical trial participation, attitude changes after the trial, willingness to participate again, and intention to recommend the trial. Participants rated each item on a 4-point Likert scale (ranging from 1 to 4), with a higher total score (ranging from 8 to 32) indicating a higher level of satisfaction with clinical trial participation.

#### Service quality in clinical trials.

The service quality in clinical trials was assessed across four domains: quality of interaction with researchers, performance procedures and results, and physical environment in clinical trials.

The quality of interaction with researchers was defined as participants’ perception of the quality of interaction between clinical trial participants and the researchers during the clinical trial process. The quality of interaction was assessed using three sub-dimensions (17 items) of the Research Participants’ Perception Survey (RPPS), comprising five dimensions [12]: 7 items on information, education, and communication; 6 items on participants’ trust in the researchers; and 4 items on the researchers’ respect for the participants. Each item was rated on a 4-point Likert scale (1 = never/no, 2 = sometimes/somewhat, 3 = usually/mostly, and 4 = always/completely), and the average score was calculated. A higher average score indicated positive perceptions of the quality of interaction with researchers in clinical trials.

The quality of physical environment in clinical trials refers to participants’ perception of the quality of physical facilities and environmental conditions throughout their participation in the clinical trial, beginning with the consent process to the actual conduct of the trial. It was measured using an instrument developed internally based on a literature review and interviews with clinical trial participants and clinical research coordinators (CRCs) [25]. This domain included 9 items that were grouped into 2 sub-domains: securing (4 items) and convenience (5 items) of the facilities, and spaces for conducting the clinical trial. Participants rated each item on a 4-point Likert scale (1 = no, 2 = somewhat, 3 = mostly, and 4 = completely), with a higher average score indicating positive perceptions of the quality of physical environment in clinical trials.

The quality of performance in clinical trials is divided into the quality of performance procedures and results. Quality of performance procedures in clinical trials comprised 18 items, including 12 and 6 items from the “informed consent” and “coordination of care” dimensions, respectively, of the RPPS [13]. Items related to whether participants felt pressured by the researcher to participate in the clinical trial or to continue if they had considered leaving the trial were converted by inverting the score. The item scale and score calculation methods were the same as those used for the quality of interaction with researchers. A higher average score indicated positive perceptions of the performance procedures in clinical trials. Quality of performance results in clinical trials was assessed using the Quality of Informed Consent (QUIC) [26]. QUIC comprises two parts: Parts A and B, which are designed to measure objective and subjective understanding, respectively. In this study, 14 items of Part B were used to measure the subjective understanding of the clinical trials as the quality of performance results in clinical trials perceived by the participants. Participants rated their level of understanding about clinical trials on a 5-point Likert scale ranging from “I did not understand this at all” (1 point) to “I understood this very well” (5 points). Each item was transformed into a score ranging from 0 to 100 using the formula “(raw score—1) × 25,” with a higher average score indicating a higher level of quality of performance results in clinical trials.

### Participant characteristics

Participant characteristics were measured in terms of demographic characteristics (7 items), health characteristics (4 items), and clinical trial participation characteristics (18 items). The specific variables are presented in Table 1.

### Data collection

Data were collected from March 29 to May 26, 2023, from adults participating in clinical trials at two hospitals affiliated with Y Medical Center located in Seoul, Korea. The data collection was conducted at Severance Hospital from March 30 to May 12, and at Gangnam Severance Hospital from April 14 to May 26. Prior to data collection, the study purpose and contents were explained to the clinical trial centers and staff, and their permission and cooperation were requested. Detailed explanations were provided to eligible participants, who were either in the waiting area or undergoing medication, regarding the study. If a clinical trial patient expressed willingness to participate in the study, the survey was initiated. Data collection was conducted via an offline paper-based questionnaire or an online survey (via Survey Monkey), and participants were given the option to choose their preferred method for the survey. The mean questionnaire completion time was 22.07±9.52 min (median 20 min), ranging from 6 to 43 min.

### Data analysis

Data analysis was performed using the SPSS Statistics program version 26.0 for Windows (IBM Corp, USA: Amonk, NY). The characteristics of Korean clinical trial participants, perceptions of clinical trial service quality, and levels of participant satisfaction in clinical trials were analyzed using descriptive statistics, such as frequencies, percentages, means, and standard deviations. Differences in service quality and satisfaction with participation in clinical trials based on the characteristics of Korean clinical trial participants were analyzed by conducting an independent t-test or one-way ANOVA (with the Scheffé post-hoc test). The correlation between perceived service quality and satisfaction with participation in clinical trials among Korean clinical trial participants was analyzed using Pearson’s correlation coefficient. Factors influencing satisfaction with participation in clinical trials were analyzed by conducting multiple linear regression analysis (following the enter method). All variables that were significantly associated with the outcome variable in the univariate analysis were entered into the regression analysis.

### Ethics approval and consent to participate

This study was conducted after obtaining approval from the Institutional Review Boards (IRBs) of the related institutions in March and April 2023 (IRB numbers: 4-2023-0090, and 3-2023-0035). Prior to conducting a survey, the study purpose and methodology were explained to all participants, and they were given the right to voluntarily participate or withdraw consent. The participants were given the informed consent form and allotted ample time to make voluntary decisions regarding their participation. All surveys were conducted after obtaining written consent from participants.

## Results

### Participant characteristics

The major demographic and health characteristics of participants are presented in Table 2. Of participants, 54.9% and 45.1% were men and women, respectively. The average age was 56.33 ± 12.83 years. Most participants (72.4%) had no or minimal difficulty affording medical expenses. Most participants (83.5%) were in anticancer clinical trials, and the remaining were in clinical trials related to musculoskeletal, endocrine, gastrointestinal, cardiovascular, kidney or rheumatic, central nervous system, respiratory, dermatological, and ophthalmic disorders. A total of 34.5% of participants had one or more comorbidities that were unrelated to their clinical trial participation, and 73.3% reported a normal or better individual subjective health status.

**Table 2:** Service quality and participant satisfaction in clinical trials based on participants’ demographic and health characteristics (N = 206).

Variables	Category	N (%) / M ± SD	Service quality								Satisfaction	
			Interaction with researchers		Physical environment		Performance procedures		Performance results			
			M ± SD	t/F/r (*p*)	M ± SD	t/F/r (*p*)	M ± SD	t/F/r (*p*)	M ± SD	t/F/r (*p*)	M ± SD	t/F/r (*p*)
Gender	Male	113 (54.9)	3.68 ± 0.34	2.87 (.092)	3.46 ± 0.44	8.93 (.003)	3.62 ± 0.34	2.59 (.109)	80.89 ± 16.01	1.71 (.192)	27.49 ± 3.63	3.88 (.050)
	Female	93 (45.1)	3.59 ± 0.39		3.24 ± 0.60		3.54 ± 0.33		83.76 ± 15.13		26.48 ± 3.65	
Age (years)		56.33 ± 12.83	3.64 ± 0.36	-0.05 (.435)	3.36 ± 0.53	0.07 (.291)	3.58 ± 0.34	-0.07 (.305)	82.19 ± 15.61	-0.16 (.021)	27.03 ± 3.66	0.14 (.039)
Education	≤ Middle school	30 (14.6)	3.70 ± 0.24	0.70 (.499)	3.43 ± 0.48	0.60 (.550)	3.53 ± 0.41	0.47 (.615)	78.57 ± 14.21	0.98 (.378)	27.40 ± 3.54	0.18 (.839)
	High school	59 (28.6)	3.66 ± 0.30		3.30 ± 0.52		3.59 ± 0.30		82.32 ± 14.20		26.95 ± 3.75	
	≥ College	117 (56.8)	3.62 ± 0.41		3.37 ± 0.55		3.60 ± 0.34		83.04 ± 16.64		26.98 ± 3.67	
Employment status	Employed	78 (37.9)	3.66 ± 0.30	0.53 (.468)	3.39 ± 0.55	0.33 (.564)	3.60 ± 0.35	0.21 (.644)	83.65 ± 14.50	1.11 (.294)	27.17 ± 3.70	0.16 (.686)
	Non-employed	128 (62.1)	3.63 ± 0.40		3.35 ± 0.52		3.58 ± 0.34		81.29 ± 16.29		26.95 ± 3.65	
Financial difficulties with medical expenses	Not at all (a)	71 (34.5)	3.70 ± 0.36	2.10 (.101)	3.46 ± 0.51	2.86 (.038)	3.64 ± 0.27	1.82 (.144)	82.60 ± 15.69	4.96 (.002) a,b>d^1)^	27.66 ± 3.69	2.25 (.083)
	Little difficult (b)	78 (37.9)	3.66 ± 0.32		3.39 ± 0.51		3.58 ± 0.36		85.37 ± 12.74		27.12 ± 3.41	
	Some difficult (c)	36 (17.5)	3.59 ± 0.39		3.20 ± 0.58		3.57 ± 0.36		80.95 ± 15.75		25.75 ± 3.54	
	Very difficult (d)	21 (10.2)	3.49 ± 0.42		3.19 ± 0.55		3.45 ± 0.40		71.09 ± 20.37		26.81 ± 4.30	
Clinical trial related diseases	Cancer	172 (83.5)	3.63 ± 0.37	1.59 (.208)	3.35 ± 0.51	0.48 (.489)	3.57 ± 0.34	2.02 (.157)	80.89 ± 15.85	7.42 (.007)	27.01 ± 3.73	0.06 (.805)
	Others	34 (16.5)	3.71 ± 0.34		3.42 ± 0.62		3.66 ± 0.34		88.76 ± 12.84		27.18 ± 3.36	
Subjective health status	Very bad	14 (6.8)	3.55 ± 0.63	2.38 (.053)	3.13 ± 0.76	2.45 (.048)	3.61 ± 0.38	2.45 (.048)	82.91 ± 24.11	0.91 (.460)	26.07 ± 4.18	3.69 (.006)
	Bad	41 (19.9)	3.52 ± 0.31		3.22 ± 0.49		3.44 ± 0.38		78.09 ± 15.83		25.51 ± 3.72	
	Normal	100 (48.5)	3.69 ± 0.34		3.44 ± 0.48		3.63 ± 0.32		83.05 ± 14.66		27.34 ± 3.29	
	Good	48 (23.3)	3.66 ± 0.33		3.37 ± 0.57		3.60 ± 0.32		83.74 ± 14.49		27.73 ± 3.87	
	Very good	3 (1.5)	3.90 ± 0.12		3.81 ± 0.23		3.67 ± 0.13		80.95 ± 16.50		31.00 ± 1.73	

M, mean; SD, standard deviation

^1)^ Scheffé test

The major clinical trial participation characteristics are presented in Table 3. Most participants (66.0%) had a duration of participation in the clinical trial of less than 1 year. Both the primary explanations for the clinical trial (75.2%) and the obtaining of consent (81.1%) were most often conducted through the CRC. The average explanation time for clinical trials before obtaining consent was 22.92 ± 14.97 min, with 60.6% of participants explaining within 30 min and 31.6% taking 30 min or more but less than 60 min. Furthermore, 47.1% experienced adverse events, and when experiencing adverse events, the level of adverse events was most often mild (28.2%). After participating in the clinical trial, the participants reported experiencing a change in health status and chose “much better” (45.0%) and “better” (33.2%) to describe the improvement. Some participants (14.5%) said that they had considered leaving the trial.

**Table 3:** Service quality and participant satisfaction in clinical trials based on participants’ clinical trial participation characteristics (N = 206).

Variables	Category	N (%) / M ± SD	Service quality								Satisfaction	
			Interaction with researchers		Physical environment		Performance procedures		Performance results			
			M ± SD	t/F/r (*p*)	M ± SD	t/F/r (*p*)	M ± SD	t/F/r (*p*)	M ± SD	t/F/r (*p*)	M ± SD	t/F/r (*p*)
Duration of participation in the clinical trial	< 1 year	136 (66.0)	3.65 ± 0.37	0.32 (.575)	3.33 ± 0.56	1.74 (.188)	3.58 ± 0.34	0.00 (.945)	82.65 ± 15.30	0.36 (.550)	26.66 ± 3.75	4.20 (.042)
	≥ 1 year	70 (34.0)	3.62 ± 0.35		3.43 ± 0.48		3.58 ± 0.34		81.28 ± 16.36		27.76 ± 3.40	
The person who mainly explained the clinical trial	Primary care doctor, etc., researcher (a)	44 (21.4)	3.74 ± 0.32	2.91 (.057)	3.55 ± 0.53	3.57 (.030) a>b^1)^	3.61 ± 0.36	0.91 (.404)	87.87 ± 12.60	3.97 (.020) a>b^1)^	27.91 ± 3.46	3.40 (.035)
	CRC (b)	155 (75.2)	3.62 ± 0.37		3.31 ± 0.53		3.58 ± 0.33		80.79 ± 15.99		26.91 ± 3.69	
	Do not know (c)	7 (3.4)	3.45 ± 0.31		3.32 ± 0.39		3.42 ± 0.36		77.30 ± 18.55		24.29 ± 2.63	
The person who obtained consent for the clinical trial	Primary care doctor, etc., researcher	32 (15.5)	3.69 ± 0.33	1.89 (.154)	3.48 ± 0.51	0.91 (.403)	3.55 ± 0.38	2.58 (.078)	86.61 ± 13.50	2.19 (.114)	27.59 ± 3.22	1.79 (.169)
	CRC	167 (81.1)	3.64 ± 0.36		3.34 ± 0.54		3.60 ± 0.32		81.65 ± 15.75		27.02 ± 3.74	
	Do not know	7 (3.4)	3.39 ± 0.40		3.37 ± 0.42		3.32 ± 0.48		74.74 ± 19.57		24.71 ± 3.04	
Time taken to informed consent		22.92 ± 14.97	3.64 ± 0.36	0.12 (.084)	3.36 ± 0.53	0.12 (.084)	3.58 ± 0.34	0.19 (.006)	82.19 ± 15.61	0.11 (.109)	27.03 ± 3.66	0.15 (.034)
Experience of adverse events	None (a)	109 (52.9)	3.72 ± 0.30	10.16 (< .001) a>c^1)^	3.47 ± 0.49	5.24 (.002)	3.64 ± 0.32	3.99 (.009) a>d^1)^	84.70 ± 13.42	4.47 (.005) a>d^1)^	27.61 ± 3.71	3.74 (.012)
	Mild degree (b)	58 (28.2)	3.65 ± 0.31		3.35 ± 0.42		3.58 ± 0.33		81.40 ± 14.81		27.05 ± 3.32	
	Moderate degree (c)	26 (12.6)	3.46 ± 0.38		3.08 ± 0.68		3.48 ± 0.32		80.01 ± 17.37		25.69 ± 3.28	
	Severe degree (d)	13 (6.3)	3.25 ± 0.61		3.10 ± 0.68		3.35 ± 0.43		68.96 ± 25.09		24.85 ± 4.22	
Changes in health status after participation in the clinical trial *	Get worse (a)	7 (3.5)	3.12 ± 0.78	6.45 (< .001) a<b,c,d^1)^	2.76 ± 0.89	6.60 (< .001) a<c,d^1)^	3.20 ± 0.33	6.45 (< .001) a<d^1)^	73.21 ± 29.27	0.98 (.404)	24.29 ± 5.31	39.60 (< .001) a,b,c<d^**1)**^
	No change (b)	37 (18.3)	3.61 ± 0.33		3.16 ± 0.58		3.52 ± 0.41		83.72 ± 14.79		24.31 ± 3.25	
	Better (c)	67 (33.2)	3.62 ± 0.36		3.36 ± 0.47		3.54 ± 0.34		81.74 ± 15.54		25.54 ± 2.99	
	Much better (d)	91 (45.0)	3.71 ± 0.30		3.48 ± 0.47		3.67 ± 0.27		82.83 ± 14.10		29.49 ± 2.48	
Considering leaving the clinical trial	No	176 (85.4)	3.68 ± 0.30	13.28 (< .001)	3.41 ± 0.51	8.86 (.003)	3.60 ± 0.34	3.05 (.082)	83.22 ± 13.69	5.37 (.021)	27.55 ± 3.44	27.21 (< .001)
	Yes	30 (14.6)	3.43 ± 0.56		3.10 ± 0.60		3.48 ± 0.36		76.13 ± 23.54		24.00 ± 3.49	

M, mean; SD, standard deviation; CRC, Clinical research coordinators

^1)^ Scheffé test

### Level of service quality and participant satisfaction in clinical trials

The perceived service quality level and participant satisfaction in clinical trials are presented in Table 4. The level of participant satisfaction in clinical trials was generally positive, with 27.03 ± 3.66 points (Mean of the 8 items was 3.39 ± 0.46, with a maximum score of 4 points).

**Table 4:** Correlations between service quality and participant satisfaction in clinical trials (N = 206).

	M ± SD (Range)	Service quality				Satisfaction
		Interaction with researchers	Physical environment	Performance procedures	Performance results	
		Correlation coefficient				
Quality of interaction with researchers	3.64 ± 0.36 (1–4)					
Quality of physical environment	3.36 ± 0.53 (1–4)	.606^3)^				
Quality of performance procedures	3.58 ± 0.34 (1–4)	.697^3)^	.498^3)^			
Quality of performance results	82.19 ± 15.61 (0–100)	.567^3)^	.392^3)^	.516^3)^		
Participant satisfaction	27.03 ± 3.66 (8–32)	.533^3)^	.491^3)^	.464^3)^	.390^3)^	

M, mean; SD, standard deviation

^1)^ significant at *p* < 0.05

^2)^ significant at *p* < 0.01, and

^3)^ significant at *p* < 0.001

The recognition of service quality in clinical trials by clinical trial participants was also generally positive. Among the domains of service quality in clinical trials measured using the same item structure, the quality of interaction with researchers was the highest at 3.64 ± 0.36 points, followed by the quality of performance procedures at 3.58 ± 0.34 points; however, the quality of physical environment was the lowest at 3.36 ± 0.53 points. Regarding the quality of interaction with researchers, the perception of researchers’ respect for the participants was the highest (3.72 ± 0.38), followed by information, education, and communication (3.64 ± 0.39), and finally, trust in clinical trial researchers (3.53 ± 0.49). By item, the highest score was obtained for whether the CRC treated participants with courtesy and respect (3.87 ± 0.38), and the lowest score was obtained for whether the treatment hours with the research doctor were sufficient (3.22 ± 0.79). Regarding the quality of physical environment in clinical trials, the perception of convenience (3.31 ± 0.59) was lower than that of securing basic facilities and space (3.43 ± 0.56). Specifically, the perception of the sufficiency of treatment and waiting spaces (3.05 ± 0.82) and the convenience of their spatial arrangement considering the movement distance (3.07 ± 0.84) were the lowest among the total service quality in clinical trials. In terms of the quality of performance procedures in clinical trials, the perceptions of the informed consent procedure (3.59 ± 0.38) and coordination of care (3.57 ± 0.41) were similar. However, the following items were evaluated much lower than the perception of the quality of the entire clinical trial performance procedures: Whether the informed consent form (ICF) prepared them for what they should expect during the trial (3.19 ± 0.70), whether the participants felt that they had enough time to think before signing the ICF after receiving the explanation about the clinical trial (3.29 ± 0.79), whether the information and explanation provided before participating in the clinical trial were helpful in the actual clinical trial process (3.30 ± 0.70), and whether they ever had to wait too long in the clinical trial process (3.29 ± 0.85 in reverse transformation). The subjective understanding of the clinical trial evaluated as the quality of the clinical trial performance was, on average, 82.19 ± 15.61 points. By detailed items, the understanding that participation in the clinical trial was voluntary was high (92.72 ± 15.44). In contrast, understanding of treatment options aside from clinical trial participation (70.75 ± 31.98) and who would cover treatment costs if they were injured or became ill because of participation (65.29 ± 33.82) was lower compared to the overall average understanding.

### Service quality and participant satisfaction in clinical trials by participant characteristics

A comparison of service quality and satisfaction by the participants’ demographic and health characteristics is presented in Table 2. In particular, age and subjective health status showed significant differences in participant satisfaction. Regarding awareness of service quality in clinical trials, the awareness of the quality of performance results decreased with increasing age, and when participants were involved in clinical trials for cancer compared to when they were not. Depending on the subjective health status, differences were observed in the level of awareness regarding the quality of physical environment and performance procedures, and participant satisfaction.

Table 3 presents a comparison between service quality and participant satisfaction based on participants’ clinical trial participation characteristics. Among these characteristics, variables that showed significant differences in participant satisfaction were the clinical trial participation period, the person who primarily explained the clinical trial, the time taken to informed consent, the experience of adverse events, health changes after participation in the clinical trial, and whether to consider discontinuing clinical trial participation. Satisfaction was higher in cases where health status had improved significantly after participation in the clinical trial (29.49 ± 2.48) than in those where health status had worsened (24.29 ± 5.31), in cases for which health status had not changed (24.31 ± 3.25), or in cases for which health status had only improved slightly (25.54 ± 2.99). Furthermore, as the time taken to obtain informed consent increased, participant satisfaction with clinical trial participation also increased when discontinuation of clinical trial participation was not considered. Regarding the quality of clinical trial services, when the main consent explainer was a researcher, such as a doctor, the level of awareness of physical environment quality (3.55 ± 0.53 vs. 3.31 ± 0.53) and performance results (87.87 ± 12.60 vs. 80.79 ± 15.99) was higher than when the explainer was the CRC; the longer the time to obtain informed consent, the higher the level of awareness of the quality of performance procedures. The perception of the quality of most clinical trial services differed significantly depending on the participants’ experience with adverse events, changes in health status after participation in the clinical trial, and their consideration of discontinuing the clinical trial.

### Correlation between service quality and participant satisfaction in clinical trials

Each domain of service quality and participant satisfaction in clinical trials showed a significant positive correlation (*p* < .001). Concerning participant satisfaction in clinical trials, the highest correlation was observed with the quality of interactions with researchers in clinical trials (r = .533), followed by physical environment in clinical trials (r = .491), performance procedures in clinical trials (r = .464), and performance results in clinical trials (r = .390; Table 4).

### Factors associated with participant satisfaction in clinical trials

Prior to conducting multiple regression analysis, we verified that the basic assumptions of the regression model were satisfied, including independence, equality of variance, and the normality of errors. This was done through residual analysis, entailing scrutiny of the residual plot and the normal probability plot. Furthermore, the variance inflation factors (VIF) were less than 10 (1.07–2.74), confirming no multicollinearity issues between the independent variables. To identify the factors affecting participant satisfaction in clinical trials, all variables that were significantly associated with the outcome variable in the univariate analysis were entered into the regression analysis. These included age, subjective health status, duration of participation in the trial, the person primarily explaining the trial, time taken to informed consent, the experience of adverse events, changes in health status after participation in the clinical trial, consideration of discontinuing the clinical trial, the quality of interaction with researchers, physical environment, and performance procedures and performance results in clinical trials.

Service quality factors that significantly predicted participant satisfaction in clinical trials included quality of interaction with researchers (β = .21, *p* = .012), physical environment (β = .12, *p* = .047), and performance results in clinical trials (β = .14, *p* = .025). Variables other than service quality included changes in health status after participating in the trial (β = .41, *p <* .001) and consideration of discontinuing the trial (β = -.14, *p* = .009). The final regression model was statistically significant (F = 18.99, *p <* .001), and it explained 54.9% (R^2^ = .549, Adjusted R^2^ = .520) of the variance in participant satisfaction in clinical trials (Table 5).

**Table 5:** Prediction of participant satisfaction in clinical trials (N = 206).

Variables	B	SE	β	t	*p*	VIF
(intercept)	5.92	3.01		1.97	.051	
Quality of interaction with researchers	2.10	0.82	.21	2.55	.012	2.74
Quality of physical environment	0.82	0.45	.12	1.84	.047	1.72
Quality of performance procedures	0.66	0.79	.06	0.83	.405	2.17
Quality of performance results	0.03	0.01	.14	2.26	.025	1.62
Age	0.02	0.02	.06	1.09	.276	1.19
Subjective health status	0.24	0.22	.06	1.08	.280	1.13
Duration of participation in the clinical trial	0.30	0.41	.04	0.74	.462	1.15
The person who mainly explained the clinical trial	-0.24	0.35	-.03	-0.68	.497	1.07
Time taken to informed consent	-0.01	0.01	-.02	-0.47	.638	1.12
Experience of adverse events	0.13	0.23	.03	0.56	.578	1.38
Changes in health status after participation in the clinical trial	1.75	0.24	.41	7.15	< .001	1.34
Considering leaving the clinical trial	-1.48	0.56	-.14	-2.63	.009	1.25

M, mean; SD, standard deviation; VIF, variance inflation factors

## Discussion

In this study, overall participant satisfaction with the clinical trial was positive, similar to previous findings by Sano et al. (total mean 27.30 ± 3.08) [8]. Despite 83.5% of participants in this study being in anticancer clinical trials, most perceived their subjective health status as normal or above. Additionally, the subjective health status of participants was identified as one of the significant factors influencing participant satisfaction in clinical trials. Considering these factors, it is plausible to speculate that among cancer patients, those with better subjective health statuses may have significantly participated in this study, potentially resulting in higher participant satisfaction ratings compared to the overall level of clinical trial participants in Korea. Therefore, caution is needed in interpreting the results.

The quality of interaction with researchers was found to be the most significant factor associated with participant satisfaction among the domains of clinical trial service quality. This aligns with previous research emphasizing the importance of the participant–researcher relationship and trust in enhancing clinical trial participant satisfaction [3, 5, 10, 11]. Providing appropriate information and building trust based on respect for clinical trial participants are also fundamental ethical principles of clinical trials [27]. Open communication that prioritizes the well-being and concerns of clinical trial participants is imperative, in that participation in clinical trials can cause anxiety and stress owing to the inherent potential risks and experimental nature of clinical trials [28]. The researchers should be able to maintain a trusting relationship through informative, friendly, and patient-centered communication with participants [11]. To achieve this, in addition to researcher initiatives to maintain superior interactions, a range of support measures for researcher education should be instituted. These include qualifications as clinical trial experts, focusing on principles of respect and accountability toward clinical trial participants, and advanced curricula to strengthen practical competencies, encompassing communication aptitude.

The quality of performance results, which measured participants’ subjective understanding of the clinical trial, was the second-most influential factor in participant satisfaction, in terms of the service quality in clinical trials. This finding is consistent with previous research that demonstrated a positive correlation between subjective understanding of clinical trials and participant satisfaction [7], while also showing a negative correlation with regretting participation in clinical trials [29]. However, in our study, the level of understanding among clinical trial participants in the current study was notably lower than that reported in studies of cancer patients in the United States (mean 81.9–90.2) and Australia (mean 91.5) [7, 29]. These findings raise concerns about whether voluntary consent in Korean clinical trials is truly obtained through adequate information provision and appropriate understanding of the trial procedures. Simultaneously, they suggest the need for strategies to enhance comprehension of clinical trials to improve participant satisfaction.

In addition, while the quality of the informed consent process, assessed as the quality of performance procedure, was not a statistically significant predictor of participant satisfaction, it demonstrated a significant positive correlation with satisfaction levels. However, the time taken to explain the trial before obtaining consent was found to be considerably shorter than that in international research findings [30]. Above all, the lower evaluation of the usefulness of information and explanations provided prior to clinical trial participation compared to the perception of the quality of the overall clinical trial performance procedure has significant implications. In clinical trials, the informed consent process is the fundamental principle protecting participants, and is particularly important for ensuring an ethical process. Many previous studies have identified long and complicated ICFs that are difficult for the public to understand as a major factor that reduces participants’ understanding [31, 32]. Thus, practical efforts are required to improve the quality of performance in clinical trials beyond providing one-sided information from the researcher’s side solely to meet legal and regulatory requirements. This can start with the basics, such as the use of ICF utilizing simplified language, concise sentences, bold text, and colors, as well as the provision of sufficient explanations and opportunities for questioning. The ICF should offer the information that the clinical trial participants need at a level that they can understand.

In this study, the quality of physical environment within clinical trials, a relatively unexplored area in previous research, was considered a significant aspect of service quality. While it was found to be significantly associated with participant satisfaction, it was rated the lowest by participants among all three domains of service quality in clinical trials. In particular, the sufficiency of treatment and waiting spaces, as well as the convenience of their spatial arrangement, received the lowest scores among all assessment areas measured using the same item structure for evaluating the overall service quality in clinical trials. According to interviews with domestic clinical trial participants and CRCs, clinical trial participants often experience longer waiting times compared to general patients owing to protocol compliance and participant monitoring. However, their primary complaints were focused on the inconvenience stemming from insufficient waiting areas. While larger hospitals allocate dedicated spaces for clinical trials, such as clinical trial centers, participant discomfort was exacerbated when examination and treatment areas for clinical trials were situated far from general outpatient areas [25]. These findings highlight the need for greater consideration of patients participating in actual clinical trials, not just researchers alone, from the establishment of basic infrastructure and the implementation of clinical trials. Moreover, considering the current concentration of clinical trials in the Korean capital, Seoul [33], significant differences may be observed in the quality of physical environment in clinical trials, specifically in terms of basic infrastructure, among hospitals and regions. Therefore, follow-up research that focuses on regional institutions conducting clinical trials, as well as research on researchers and participants, is needed to better understand the specific characteristics related to the clinical trial environment.

### Limitations

This study is meaningful as it takes an additional step in safeguarding the basic rights of clinical trial participants and provides fundamental data for establishing strategies to conduct clinical trials with high satisfaction from the perspective of clinical trial participants. Nevertheless, the study had several limitations that should be considered when interpreting the findings and planning future research. First, regarding research methodology, the data collection process centered on clinical trial participants from two tertiary hospitals in Seoul and relied on convenience sampling. Consequently, caution must be exercised when generalizing or extrapolating the results derived from this cross-sectional survey study. Specifically, 83.5% of the individuals in this study were participants in anticancer clinical trials. According to the status of investigational new drug approvals, over 30% of clinical trials involve anticancer drugs, while cardiovascular and endocrine drug trials constitute over 10% of such endeavors in Korea [34]. Thus, it is imperative to conduct further replication and follow-up studies, considering participants’ health characteristics and the types of clinical trials in which they are involved. Additionally, given that Hospital A, where data collection was primarily conducted, is one of the highest-level hospitals conducting most clinical trials in Korea, it is necessary to consider the potential differences in the service quality and satisfaction of clinical trials depending on the environment in which they are conducted or the expertise of personnel.

## Conclusion

In this study, clinical trial participants’ perceptions of the service quality of clinical trials and their satisfaction with participation were generally positive. Participants’ positive perceptions of each service quality domain in clinical trials were found to have a significant correlation with and predicted increased satisfaction with participation. It is crucial to evaluate participants’ experiences throughout the clinical trial process, considering components of service quality from the clinical trial participant’s perspective. Accordingly, satisfactory interactions with clinical trial participants must occur to ensure high-quality clinical trial performance and environmental quality. This requires individual efforts by researchers as well as organizational and institutional interventions and support. This will contribute to increasing satisfaction and strengthening the competitiveness of clinical trial participation by providing high-quality services in terms of clinical trial participants. In future research, quantitative studies should be conducted to examine the experiences, satisfaction levels, and adherence rates of clinical trial participants from diverse backgrounds and environments. Additionally, qualitative research, such as individual or focus group interviews, should be undertaken to gain a deeper understanding of participants’ experiences and to explore practical improvements in clinical trial processes.
