# Expression of Concern: Improving patient satisfaction based on service quality in clinical trials: A cross-sectional study

**DOI:** 10.1371/journal.pone.0357254

**Published:** 2026-08-31

**Authors:** The PLOS One Editors

## Notice of Republication

This article was republished on April 28, 2026, to address an issue identified post-publication. Please download this article again to view the correct version.

The article’s Data Availability statement is updated to: The dataset underlying this study cannot be made publicly available due to ethical restrictions specified in the IRB-approved informed consent form under which the data were collected. The data include demographic and clinically relevant participant information; while identifying information has been coded, the possibility of re-identification cannot be entirely ruled out when combined with additional information. The consent form limits the use of the data to the purposes of the original study, prohibits provision to any third party, and specifies the permanent destruction of the data after the mandatory retention period. Inquiries regarding the permitted scope of use of these data, or regarding the related ethics records, may be directed to the Institutional Review Board of Severance Hospital, Yonsei University Health System (irb@yuhs.ac).

The first author stated that the original raw data files supporting the article’s results were permanently destroyed on July 1, 2026.

As the original raw data files supporting the article’s results are no longer available, this article [1] does not comply with the PLOS Data Availability policy. Therefore, the *PLOS One* Editors issue this Expression of Concern.
